# A Case of Uncomplicated Bacteremia Caused by Capnocytophaga canimorsus in an Immunocompetent Patient

**DOI:** 10.7759/cureus.44293

**Published:** 2023-08-28

**Authors:** Daisuke Taniyama, Kazuya Imoto, Michio Suzuki, Koichi Imaoka

**Affiliations:** 1 Department of Infectious Diseases, Showa General Hospital, Tokyo, JPN; 2 Department of General Internal Medicine, Saiseikai Yokohamashi Tobu Hospital, Yokohama, JPN; 3 Department of Veterinary Science, National Institute of Infectious Diseases, Tokyo, JPN

**Keywords:** dog, animal bite, immunocompetent patients, uncomplicated bacteremia, capnocytophaga canimorsus

## Abstract

This report describes uncomplicated bacteremia caused by *Capnocytophaga canimorsus *in an immunocompetent woman who presented with rigor and fever. She was hemodynamically stable. Two blood samples were immediately cultured because rigor indicated bacteremia. Although her symptoms were relieved, Gram-negative rods grew from blood cultures. She noted that she had been bitten by her dog before the first examination. The bacterium was confirmed as *C. canimorsus* by gene analysis. Infection with *C. canimorsus* can be fatal when accompanied by sepsis in elderly or immunocompromised patients. However, this case was considered rare as the patient was 41 years old and immunocompetent.

## Introduction

*Capnocytophaga canimorsus* is commonly present in the normal flora of dogs and cats. Consequently, *C. canimorsus *infection primarily arises from animal bites inflicted by these animals. It may present with severe sepsis or septic shock with organ dysfunction, most frequently coagulopathy and acute kidney injury in elderly patients and immunocompromised patients. Although the overall mortality rate was reported to be approximately 30 % [1,2], in 2016 Hästbacka et al. reported that the mortality associated with bacteremia caused by *C. canimorsus* was 5% [3]. The mortality is lower than that previously reported. In Japan, from 1993 to the end of 2017 *C. canimorsus* was found in just 88 patients with 18 deaths which was confirmed by the Ministry of Health, Labor and Welfare of Japan [4]. We report herein a rare case of uncomplicated bacteremia caused by *C. canimorsus*. The aim of this report is to describe a case of uncomplicated bacteremia caused by *C. canimorsus* in a 41-year-old immunocompetent patient.

## Case presentation

**Figure 1:**
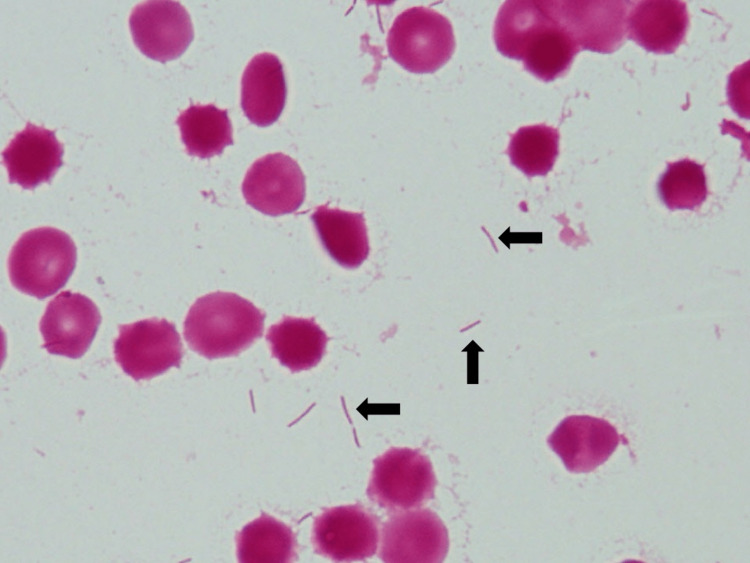
Gram stain of blood culture The image shows Gram-negative, filamentous bacilli (arrow).

A 41-year-old woman presented to the emergency department with sudden left-sided chest pain, rigor, and fever. She had no past medical history, and her past surgical history includes removal of ovarian cysts 10 years ago. She was neither a social drinker nor a steroid user. Twelve hours before visiting our hospital, she was aware of left front chest pain, which she described as being similar to muscle pain without particular incentives. Initially, the pain was blunt, but it gradually worsened, and by bedtime, she could not move because of the pain. The next morning, her left chest pain persisted and was accompanied by a fever and rigors which prompted emergency room (ER) visit. On arrival in the ER, the Glasgow Coma Score was 15 (eye opening 4, best verbal response 5, best motor response 6), temperature was 37.9°C, blood pressure was 110/76 mmHg, heart rate was 118 beats/min, respiratory rate was 18 breaths/min, and she exhibited no remarkable findings. However, she had a slight tenderness on the subclavian (a range of approximately 10 × 10 cm). Her leukocyte cell and C-reactive protein counts were 18940 /μL (reference values 4000-10000/μL) and 1.52 mg/dL (reference values 0-0.5 mg/dL), respectively, and Howell-Jolly body was not seen. Later, further tests were done to confirm her immune status. The HIV (human immunodeficiency virus) test was negative and complement C3, complement C4, and hemolytic complement activity (CH50) were in the normal range. Chest X-ray revealed no remarkable findings and electrocardiography findings revealed only sinus tachycardia without ST-T change. Chest computed tomography revealed no pneumonia and her spleen was normal. Two blood samples for culture were taken immediately because rigor may be related to bacteremia. She required follow-up in the outpatient department because she declined admission. Antibiotics were not started, as her condition was well. Her body temperature dropped from 37.9°C to 36.4°C on the day after the first examination, and her symptoms were relieved. She visited our hospital three days later and reported that she had been bitten by her pet dog on her right middle finger six days before ER visit. The swelling of the bitten part was completely resolved in a few days, and there were no remarkable findings at her first visit. However, Gram-negative rod bacteria grew from her blood cultures just after she went home on day three (Figure 1).

**Figure 2:**
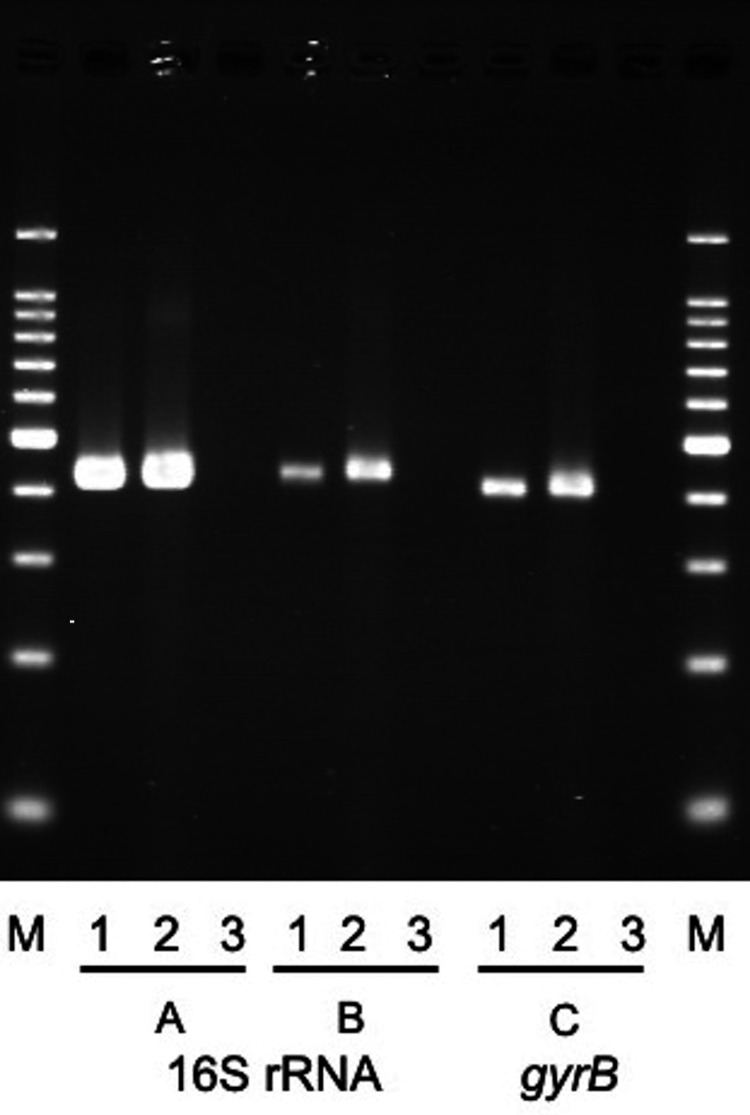
Specific detection of Capnocytophaga canimorsus 16S rRNA and gyrB gene sequences using polymerase chain reaction 1: Positive control; 2: Our case; 3: Negative control

Two sets of anaerobic bottles became positive 69 and 73 hours after the blood culture collection; one set of aerobic bottles became positive 125 hours later by BACT/ALERT®. The bacterium was a filamentous and elongated Gram-negative bacillus; it was difficult to estimate anything more. Antibiotics were withheld until the bacterium was identified, as the patient remained stable. On the 12th day after the first visit, the bacterium was identified as *Capnocytophaga canimorsus *using the HN-20 Rapid 'Nissui' identification kit (Nissui Pharmaceutical Co., Ltd., Tokyo, Japan). As there were no local symptoms or findings of bacteremia in her body, she was treated with amoxicillin 2 g/day for 14 days to prevent recurrence of bacteremia and complications. The patient remained well with no recurrence of *C. canimorsus* infection. After that, the isolate was identified using polymerase chain reaction (PCR). This method targets 16S rRNA and the *gyrB* gene and can specifically detect only *C. canimorsus*. Since a specific band was observed in all of the primers (A, B, and C) in this case, it was confirmed that the isolated bacterium was *C. canimorsus* (Figure 2). 

The antimicrobial susceptibility in the disk diffusion method is shown in Table 1.

**Table 1:** Antimicrobial susceptibility test for isolated Capnocytophaga canimorsus PCG: Penicillin G; AMPC/CVA: Amoxicillin/Clavulanate; CTRX: Ceftriaxone; IPM: Imipenem; GM: Gentamicin; MINO: Minocycline; CPFX: Ciprofloxacin

Antimicrobial	Disk (mm)	E test (μg/ml)
PCG	27	0.19
AMPC/CVA	32	0.25
CTRX	25	1.5
IPM	40	0.38
GM	-	> 256
MINO	42	0.047
CPFX	27	0.38

## Discussion

There are approximately 4000 canine bite accidents annually in Japan [5]. As this is only the number reported to the public health center, it is presumed that the total number of canine bite accidents was much higher. It is important to recall microorganisms, for example, *Pasteurella* spp., *Bartonella* spp. (Cat scratch disease), *C. canimorsus*, rabies virus, etc if we are bitten by dogs. As patients infected with *Bartonella* rarely die and domestic rabies cases have not been reported since 1956 in Japan, *C. canimorsus* infection might be one of the most important bacterial infections associated with bite accidents involving dogs. 

Two unique factors were identified in the current case, even though it was a case of uncomplicated bacteremia caused by *C. canimorsus*. First, *C. canimorsus* infection can present as uncomplicated bacteremia. Although *C. canimorsus* infection is infrequent, it commonly clinically presents as a disease that follows a serious event [6]. To date, most of the reports on *C. canimorsus* infection have found that sepsis is common and mortality is approximately 30% [1,2]. Publication bias is the likely factor making it difficult to report cases that did not follow a serious course. Additionally, the diagnosis method for this disease might also be involved. Despite infections caused by animal bites, this disease is not likely to create lesions in the wound site; bacteria are most commonly detected using blood cultures [7]. Blood cultures are often taken for hospitalization. However, for less serious cases, blood cultures may not be requested and thus this disease may not be diagnosed. In the current case, although the patient did not agree to hospitalization, the ER doctor suspected bacteremia from rigor. Thus, blood cultures were taken which led to the diagnosis of *C. canimorsus *infection. It is believed that many of the cases of *C. canimorsus* infection are never diagnosed as blood cultures are not always taken [8]. As reported in this case, the animal bite was only revealed later, highlighting the importance to investigate the contact history with animals when diagnosing patients with fever whose source of infection is unknown. If there is history of contact with animals, blood cultures should be taken.

The second interesting observation in this case was that this disease can also occur in immunocompetent patients. Historically, it is known that this disease most frequently occurs in elderly patients or those with underlying diseases. It was reported that the average age of the 93 cases in Japan from 1993 to 2017 was 64 years old [4]. Diabetes mellitus, asplenia, splenectomy, and drinking alcohol have also been reported as underlying causes or risk factors for this disease [3,9,10,11]. In the current case, the patient was just over 40 years of age, and she neither had diabetes mellitus nor drank alcohol. Additionally, her blood test is not in keeping with the immunocompromised host and her spleen showed no morphological abnormality. Taking these factors into consideration, this was a case of bacteremia caused by *C. canimorsus* in an immunocompetent patient, explaining why she did not have a serious clinical course. Hästbacka et al. reported that the mortality associated with bacteremia caused by *C. canimorsus* was 5% and of the serious cases treated in the intensive care unit mortality was 19%. This rate is similar to that of the conventionally reported mortality rates [3]. Moreover, in this report, there were 4.1 *C. canimorsus* cases estimated per 1 million population. This incidence is much higher than the incidence of 0.5-0.7 persons per 1 million population reported in earlier epidemiological surveys [10,12]. In the study conducted by Hästbacka et al., the mortality declined because it was possible to diagnose mild cases that could not be previously diagnosed. Additionally, mortality among severe cases is similar to that in conventional reports, and the bacteremia caused by *C. canimorsus* reported to date might have been more common in severe cases. 

It is believed that more non-severe, uncomplicated bacteremia cases caused by *C. canimorsus* exist. In Japan, the mortality caused by *C. canimorsus* bacteremia was 20.5 % (18 death of 88 cases) confirmed from 1993 to the end of 2017 by the Ministry of Health, Labor and Welfare [4]. Septic shock is the most frequent *C. canimorsus* presentation reported in Japan. Therefore, it is believed that the *C. canimorsus* bacteremia reported in the past may have been biased toward severe cases, suggesting that the true clinical manifestation of bacteremia caused by *C. canimorsus* may be misrepresented in Japan. Due to the widespread use of matrix-assisted laser desorption ionization-time of flight mass spectrometry, this diagnostic tool will increase the number of detections in the future, and thus it might be able to detect mild cases like uncomplicated bacteremia. The current case of uncomplicated bacteremia caused by *C. canimorsus*, which did not follow a serious clinical course, may be the first step toward grasping the clinical manifestation of *C. canimorsus* bacteremia. 

In this case, the reason why she was not severe may be that she had been overt or subclinical infection. In our case, if there was overt or subclinical infection of *C. canimorsus* infection, it is possible that the immune system responded quickly to the *C. canimorsus* infection preventing serious onset. In order to prove this, a *C. canimorsus* antibody test would be required; however, the test may have cross-reactivity with bacteria that cause periodontal disease. Additionally, it has not been established yet. Therefore, in this case, it was not possible to prove that the patient was affected by *C. canimorsus* infection before this episode. In the future, a *C. canimorsus* antibody test should be developed. 

The clinical features of *C. canimorsus* infection are not well understood because it has only been 40 years since the first case was reported. Additionally, there is no surveillance system for the reporting of such cases. Most of the reports to date have detailed patients with serious and fatal clinical courses, creating the illusion that *C. canimorsus* infections have such clinical features. It is important to recognize cases that do not follow a serious clinical course as well in order to understand all the clinical features of *C. canimorsus* infection. Future studies need to determine why some cases do not follow a serious clinical course, thus contributing to a decrease in the mortality associated with *C. canimorsus *infection and to show true clinical features of *C. canimorsus* infection.

## Conclusions

We report herein a rare case of uncomplicated bacteremia caused by *C. canimorsus*. Bacteremia, although almost always a medical emergency, may not always lead to life-threatening complications like multiple-organ failure. Clinicians should take blood cultures when diagnosing patients with fever whose source of infection is unknown, especially if there is history of contact with animals. These practices will help to figure out true clinical features of *C. canimorsus* infection.
